# A novel method to simulate radiographs of 3D printed objects

**DOI:** 10.1002/acm2.70159

**Published:** 2025-08-03

**Authors:** Maxwell C. Campbell, Steven I. Pollmann, Jaques S. Milner, Emily A. Lalone, David W. Holdsworth

**Affiliations:** ^1^ School of Mechanical and Materials Engineering Western University London Ontario Canada; ^2^ Imaging Research Laboratories Robarts Research Institute London Ontario Canada; ^3^ School of Biomedical Engineering Western University London Ontario Canada; ^4^ Department of Medical Biophysics Western University London Ontario Canada

**Keywords:** 3D printing, radiography artifact, fused deposition modeling

## Abstract

**Background:**

3D printing has a number of applications within medicine and healthcare. In applications involving radiography, the internal infill structure and external geometry of a 3D printed part can produce undesirable artifacts, limiting the full potential of 3D printing as a manufacturing technology. While the mechanical performance of a 3D printed part can be easily simulated, it is difficult to simulate the radiographic artifact produced.

**Purpose:**

The purpose of this work was to develop a tool that allows users to simulate the radiographic artifact produced by a 3D printed object.

**Methods:**

Three regular hexagons of identical geometry were sliced and 3D printed using polylactic acid (PLA) filament on a fused deposition modeling (FDM) 3D printer with varying infill patterns: rectilinear grid, cubic, and gyroid. The hexagons were then radiographed using clinical‐standard scanning protocols. The captured radiographs were compared to simulated radiographs generated using the G‐Code developed when the objects were sliced. The physical and simulated virtual radiographs were compared to one another, and the simulated angle of least and greatest artifact was noted.

**Results:**

Strong visual agreement was found between the physically captured and simulated virtual radiographs. The projection angles that produced the least amount of artifact were 22.5°, 22.5°, and 12.25° for grid, cubic, and gyroid infills, respectively. The projection angles that produced the greatest amount of artifact were 0°, 45°, and 45° for grid, cubic, and gyroid infills, respectively.

**Conclusions:**

This work provides designers of 3D printed components with a new way to evaluate a design's radiographic performance. Previously, designers would have to physically print and radiograph a part to determine the artifact produced. This work outlines the development of a tool that simulates the radiograph of a 3D printed part from multiple different projections, saving designers time to iterate to their final design.

## 1 INTRODUCTION

Three‐dimensional (3D) printing is quickly becoming an essential manufacturing technique for a wide range of industries, including medicine and healthcare.^1^ Due to the unique additive nature of 3D printing, complex part geometries that were previously impossible with traditional subtractive manufacturing techniques are now possible. As the technology matures, various applications within healthcare are now being realized, opening doors to patient‐specific medicine through bespoke surgical orthopedic implants and custom‐made surgical guides.^2^, ^3^, ^4^ There are multiple different 3D printing techniques, including stereolithography and selective laser sintering, with one of the most common and disseminated forms of 3D printing being fused deposition modeling (FDM).^5^ FDM involves the successive extrusion of semi‐molten filament material to build the part layer‐by‐layer. The designer can control the quality of the final part through the selection of printing parameters such as layer height, printing speed, and nozzle temperature.^6^ Unlike traditional subtractive manufacturing techniques, the designer can also control the mechanical properties of the final part by controlling the density and pattern of the infill used.^7^ Infill is the internal structure that provides structural integrity to the final part. The designer must balance the quality, strength, and print efficiency when selecting the printing parameters.

There are a number of applications where 3D printing is enabling advances in medical imaging; the 3D printing workflow efficiently incorporates into the CT segmentation workflow, allowing for the creation of patient‐specific phantoms,^8^, ^9^ tangible 3D printed models help improve learners’ confidence in diagnosing complex fractures,^10^ and preoperative planning.^11^ However, applications where 3D printing and medical imaging overlap create a unique set of complications. Common 3D printing materials are developed for industrial applications and have been adopted for use in medical applications. Although these materials may have suitable mechanical and thermal properties, they typically do not have ideal properties for applications in medical imaging; in protection radiography and computed tomography, one of these critical properties is the x‐ray attenuation coefficient of the material. A uniformly dense and thick material within an x‐ray field will uniformly attenuate the x‐ray beam; these artifacts can often be filtered out of the medical image with minimal loss to the signal; Neumann et al. have demonstrated this phenomenon in solid (i.e., 100% infill) polylactic acid (PLA) FDM printed rectangular specimens in a CT field.^12^ Typically, fused deposition modelled 3D printed specimens do not possess uniform cross‐sectional material densities due to differences in infill pattern and density layer‐by‐layer. The inhomogeneity in infill density in FDM 3D printed specimens creates an attenuation pattern which is more complicated to filter out, which can leave an artifact within the radiograph.^13^

Designers can work to minimize the artifacts produced by FDM 3D printed specimens by manipulating both the parts' external geometry and infill density and pattern in regions where the specimen may interfere with the anatomy being radiographed. Common open‐source slicers allow designers to define specific infill properties for subregions of an overall part, with ultimate control attainable through editing the raw g‐code. The artifact that is produced by different infill patterns can be non‐obvious. Due to the time and material constraints of 3D printing, it can be costly to print and physically radiograph specimens to ascertain the artifact produced by various structures, at multiple view angles. Our research group has developed a tool that simulates radiographs of FDM‐printed parts from G‐code, which can be used to simulate the radiographic artifact. This communication aims to examine the accuracy of this tool at simulating radiographs of simple geometries with various infill patterns and to determine which angle (between the central ray and 3D printed geometry) produces the least and greatest artifact.

## 2 METHODS

Regular hexagonal test specimens were prepared within SolidWorks (Dassault Systèmes, Waltham, MA, USA) and exported as .stl files, and sliced using Prusaslicer Version 2.6.1 (Prusa Research, Prague, Czech Republic). Three different infill patterns were examined within this communication: rectilinear grid, cubic, and gyroid; each infill pattern was printed at a density of 15%. This level of infill density was found to produce the required structural stiffness. To develop the simulated radiographs, G‐code was exported from Prusaslicer and processed with a custom software tool using VTK (Kitware Inc.; Clifton Park, NY, USA), which used the G‐code instructions to replicate the printed structures within a virtual binary image volume; this utility essentially “printed” the plastic structure as a digital volume within computer memory. The 3D binary image volumes were produced with an isotropic voxel size of 112.5 µm. ITK (Kitware Inc.) was used to mimic the x‐ray geometry of the physical radiographs, using an SID of 100 cm with a pixel spacing of 150 µm to replicate the physical scanning protocol. Digitally reconstructed radiographs (DRRs) were made for each infill pattern by forward projecting through the 3D binary image volumes at projection angles from 0°–360° in one‐degree increments.

To examine the accuracy of the simulated radiographs, regular hexagonal specimens of varying infill patterns were printed and radiographed. The hexagons were printed on a Creality Ender 3 V2 (Shenzhen Creality 3D Technology Ltd.; Shenzhen, China) with a standard 0.4 mm nozzle diameter and 0.2 mm layer height, using 1.75 mm PLA filament (Shenzen Eryone Technology Co., Ltd.; Shenzhen, China).

**FIGURE 1:**
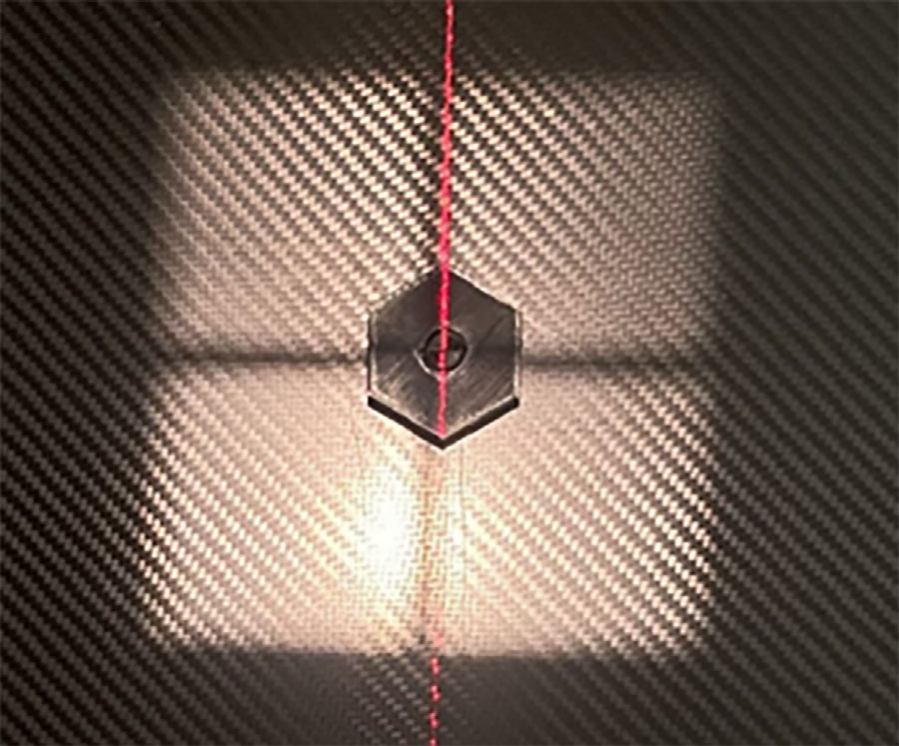
Radiographic orientation of hexagonal specimens relative to the central ray.

Each regular hexagon was radiographed using a GE Proteus XR/a x‐ray system (GE Healthcare; Milwaukee, WI, USA) with a Carestream DRX 4343 detector (Carestream; Rochester, NY, USA). A standardized source image receptor distance (SID) of 100 cm was used with the regular hexagons placed flat on the image detector such that the hexagonal face was perpendicular to the incident beam (Figure 1). The central ray beam was centered about the geometric center of the hexagonal specimen, with exposure parameters of 2.00 mAs and 55 kV to replicate clinical parameters. Raw DICOM files were imported into ImageJ, Version 1.53q (National Institutes of Health; Bethesda, MD, USA) for image analysis.

Due to the nature of this study, statistical analyses were not conducted. Rather, simulated and physical radiographs at 0° angulation (i.e., central ray perpendicular to the hexagonal face) were visually compared for agreement. This orientation was selected for comparison as this is the extruded direction of the part. As well, the angle at which the least and largest artifact occurs was determined for each infill pattern from the DRRs.

## 3 RESULTS

**FIGURE 2:**
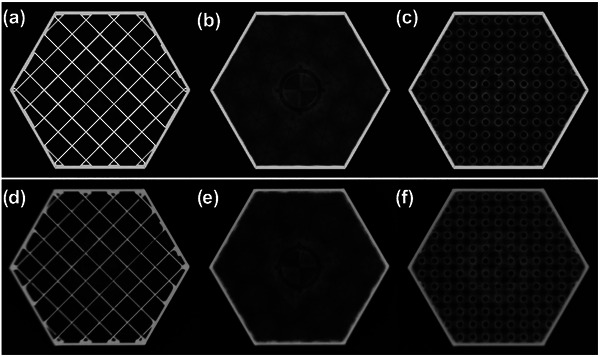
Simulated radiographs of (a) grid infill, (b) cubic infill, and (c) gyroid infill, and physical radiographs of (d) grid infill, (e) cubic infill, and (f) gyroid infill.

There was satisfactory visual agreement between the simulated and physical radiographs of the 3D printed hexagons (Figure 2).

**FIGURE 3:**
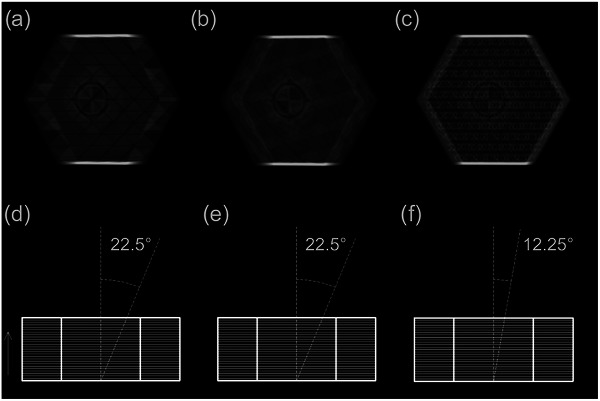
Minimum artifact of (a) grid infill and (b) cubic infill, and (c) gyroid infill. These artifacts occur at angles (d) 22.5°, (e) 22.5°, and (f) 12.25° between the extruded direction and central ray for the grid, cubic, and gyroid infill patterns, respectively. The arrow indicates the extruded direction.

The angles which produced the minimum artifact were 22.5°, 22.5°, and 12.25° for grid, cubic, and gyroid infills, respectively (Figure 3).

**FIGURE 4:**
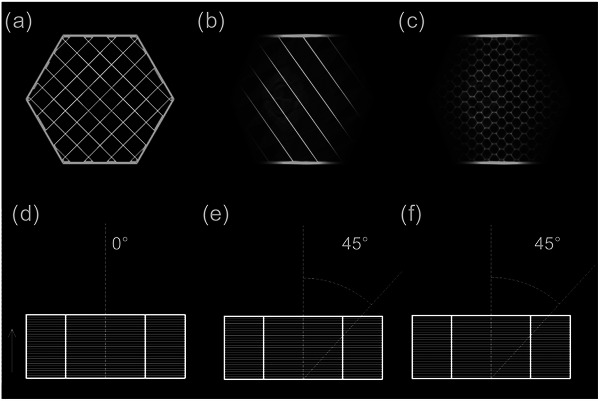
Maximum artifact of (a) grid infill, (b) cubic infill, and (c) gyroid infill. These artifacts occur at angles (d) 0°, (e) 45°, and (f) 45° between the extruded direction and central ray for the grid, cubic, and gyroid infill patterns, respectively. The arrow indicates the extruded direction.

It was found that the angle which produced the greatest artifact within the rectilinear grid infill pattern was 0° with respect to the extruded direction (Figure 4a,d). An angle of 45° between the extruded direction and the central ray produced the largest artifact in both the cubic infill and gyroid infill (Figure 4b,e & Figure 4c,f).

## 4 DISCUSSION

Our study aimed to examine the accuracy of simulated radiographs at recreating the internal geometry of FDM 3D printed parts with various infill patterns and determine the angle between the central ray and 3D printed geometry that produces the greatest artifact. Satisfactory visual agreement was found between the simulated and physical radiographs for all infill patterns considered. In simulated radiographs, we determined that the angle of greatest artifact occurred at 0° for grid infill patterns and 45° for gyroid and cubic infill patterns. This result is to be expected, as these angles correspond to the direction of infill unit cell propagation.

The greatest attenuation, and thus artifact, would occur for the ray path which passes through the greatest amount of material. This occurs when printed geometry, whether internal or external, aligns with the frustum of the beam. This is why vertical edges, both external walls and rectilinear infill patterns, such as a grid, appear with such high contrast at 0° angulation between the central ray and the extruded direction. This artifact can be reduced by avoiding perfectly vertical surfaces when the morphology of the design permits. Of the three infill patterns considered within this study, the infill pattern with the least artifact at 0° angulation was the gyroid infill pattern. The gyroid infill is comprised of a lattice of regularly repeating unit cells propagated in the *x*, *y*, and *z* directions of the 3D printed specimens. Due to the definition of a gyroid, being a periodic minimal surface with no straight lines or plane lines of curvature,^14^ there is a high level of overlap between successive layers of infill, creating a near uniform attenuation across the specimen's cross‐section compared to rectilinear patterns such as the grid or cubic patterns. Bedwani et al. found similar results, mitigating the CT artifact of 3D printed head supports by utilizing gyroid infills and non‐linear edges in their design.^13^

The limitations of our study are that only three infill patterns were considered and that the radiographed specimens are not necessarily representative of clinical objects. For the sake of brevity, we chose to only illustrate three of the most common infill patterns, representative of typical infill patterns used in industrial applications. Future work surrounding this tool should include quantifying the radiographic appearance of various infill patterns and densities.

In this report, we present a tool that designers can use to reduce the time to iterate between concepts for applications where 3D printing and radiography overlap. Currently, the design process involves modeling, printing, and radiographing parts to discern the artifact produced. This process is inherently time‐consuming. Simulating radiographs eliminates the need to physically print concepts, streamlining the design process. Further, it can be difficult to predict the artifact a 3D printed specimen will produce, especially when the infill pattern is not rectilinear, such as the case with gyroid infill patterns, when the specimen has complex geometry, or when the central ray is not perpendicular to the extruded direction. Simulated radiographs in these circumstances would reduce any ambiguity in artifacts, potentially accelerating concept generation. By understanding the interaction between the incident x‐ray beam and the deposited filament pattern within a specimen, designers could theoretically leverage infill overlap to produce uniform radiographic attenuation across the volume of a specimen. Until filaments optimized for radiographic properties are produced, this is the most likely tool a designer has to use to attempt to achieve isodensity with the anatomy of interest.

## 5 CONCLUSIONS

Our study produced acceptable visual conformity between simulated and physical radiographs of hexagonal specimens with varying infill patterns using standard clinical protocols. Our results indicated that the angle of the greatest artifact occurs at 0° for grid infills and 45° for gyroid and cubic infill patterns. The results of this study motivate designers of 3D printed parts to minimize infill density, thus reducing the artifact produced by the infill, which further has the effect of reducing overall print time. Future work includes simulating other infill patterns and densities to optimize the printing settings to minimize the appearance of the artifacts created. The applications of 3D printing within biomedical imaging have yet to be fully realized. Simulated radiographs of 3D printed specimens can help accelerate the design of various devices, from blood vessel phantoms to radiographic positioning aids, allowing medical imaging to harness the benefits of additive manufacturing.

## AUTHOR CONTRIBUTIONS

Maxwell C. Campbell, Emily A. Lalone, and David W. Holdsworth completed the research design. Maxwell C. Campbell, Steven I. Pollmann, Jaques S. Milner, Emily A. Lalone, and David W. Holdsworth completed data acquisition. Data and statistical analyses were completed by Maxwell C. Campbell. Maxwell C. Campbell, Emily A. Lalone, and David W. Holdsworth completed data interpretation. Maxwell C. Campbell wrote the manuscript. Paper critically analyzed by Emily A. Lalone, and David W. Holdsworth. All authors have read and agreed upon the final version of the manuscript.

## CONFLICT OF INTEREST STATEMENT

The authors declare no conflicts of interest.
